# Topological Data Analysis Reveals Robust Alterations in the Whole-Brain and Frontal Lobe Functional Connectomes in Attention-Deficit/Hyperactivity Disorder

**DOI:** 10.1523/ENEURO.0543-19.2020

**Published:** 2020-05-12

**Authors:** Zeus Gracia-Tabuenca, Juan Carlos Díaz-Patiño, Isaac Arelio, Sarael Alcauter

**Affiliations:** 1 Instituto de Neurobiología, Universidad Nacional Autónoma de México, Querétaro 76230, Mexico; 2 Instituto de Matemáticas, Universidad Nacional Autónoma de México, Querétaro 76230, Mexico

**Keywords:** attention-deficit/hyperactivity disorder, functional connectivity, persistent homology, resting-state fMRI, topological data analysis

## Abstract

Attention-deficit/hyperactivity disorder (ADHD) is a developmental disorder characterized by difficulty to control the own behavior. Neuroimaging studies have related ADHD with the interplay of fronto-parietal attention systems with the default mode network (DMN; Castellanos and Aoki, 2016). However, some results have been inconsistent, potentially due to methodological differences in the analytical strategies when defining the brain functional network, i.e., the functional connectivity threshold and/or the brain parcellation scheme. Here, we make use of topological data analysis (TDA) to explore the brain connectome as a function of the filtration value (i.e., the connectivity threshold), instead of using a static connectivity threshold. Specifically, we characterized the transition from all nodes being isolated to being connected into a single component as a function of the filtration value. We explored the utility of such a method to identify differences between 81 children with ADHD (45 male, age: 7.26–17.61 years old) and 96 typically developing children (TDC; 59 male, age: 7.17–17.96 years old), using a public dataset of resting state (rs)fMRI in human subjects. Results were highly congruent when using four different brain segmentations (atlases), and exhibited significant differences for the brain topology of children with ADHD, both at the whole-brain network and the functional subnetwork levels, particularly involving the frontal lobe and the DMN. Therefore, this is a solid approach that complements connectomics-related methods and may contribute to identify the neurophysio-pathology of ADHD.

## Visual Abstract

**Figure:**
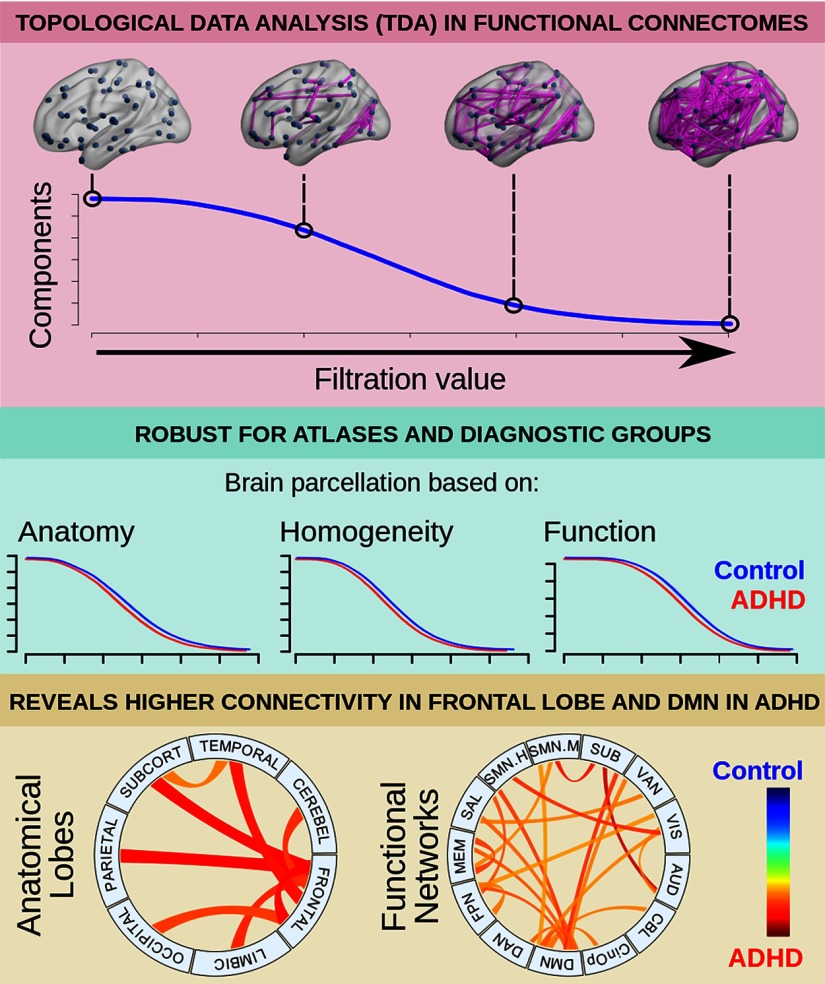

## Significance Statement

Topological data analysis (TDA) investigates the topology of interacting nodes. It may model the connectomes as a topological process instead of a static graph, exploring the transition of all nodes being isolated to binding together, as a function of the connectivity threshold. Here, we explored three parameters to characterize the algebraic topology of individual connectomes using four different brain atlases, further exploring the subnetwork levels. Our findings showed that the area under the curve (AUC) robustly differentiates children with attention-deficit/hyperactivity disorder (ADHD) and typically developing children (TDC), suggesting decreased functional segregation, with the greatest effects on the frontal lobe and the default-mode network. Overall, these results support the use of the proposed methods to robustly explore topological differences in the brain connectome.

## Introduction

Current neuroimaging technology allows the exploration of the human brain as a network of structurally and/or functionally connected constituents, i.e., voxels or regions of interest. In particular, functional connectivity is defined as the synchrony of neuronal activity patterns of anatomically separated brain regions (Aertsen et al., 1989; Friston et al., 1993) and many studies have explored this property to provide new insights about the functional organization of the brain in health and disease (van den Heuvel and Pol, 2010; Lee et al., 2013; Lord et al., 2017), providing the means to study the neurofunctional alterations of neurologic and psychiatric disorders from a systems perspective.

One of the most commonly used frameworks to explore the functional brain network is graph theory, which provides a theoretical basis to describe and characterize complex networks (Rubinov and Sporns, 2010; Fornito et al., 2013). In this framework, the brain network is modeled as a graph composed of a set of nodes (mainly voxels or larger regions) and their connections (in this case, the functional connectivity between pairs of elements). In practice, this is constructed using a matrix where each entry is a measure of connectivity between two nodes and then a threshold is applied to construct an adjacency matrix which represents the non-spurious connections. However, there is no general criterion to assign an appropriate set of regions of interest (ROIs), nor a defined threshold, which may result in divergent results among studies. For instance, several studies exploring the functional connectome of children diagnosed with attention-deficit/hyperactivity disorder (ADHD), have reported different results. Specifically, some studies have found higher network segregation and lower integration in ADHD patients compared with controls (Wang et al., 2009b; Lin et al., 2014), while others found no differences when exploring the same properties (Cocchi et al., 2012; Sato et al., 2013). Such divergent results may be partially explained by the variability in methods, including threshold and ROIs selection (Konrad and Eickhoff, 2010; Castellanos and Aoki, 2016), as well as the variable robustness of some of the most used approaches (Somandepalli et al., 2015).

**Figure 1.**
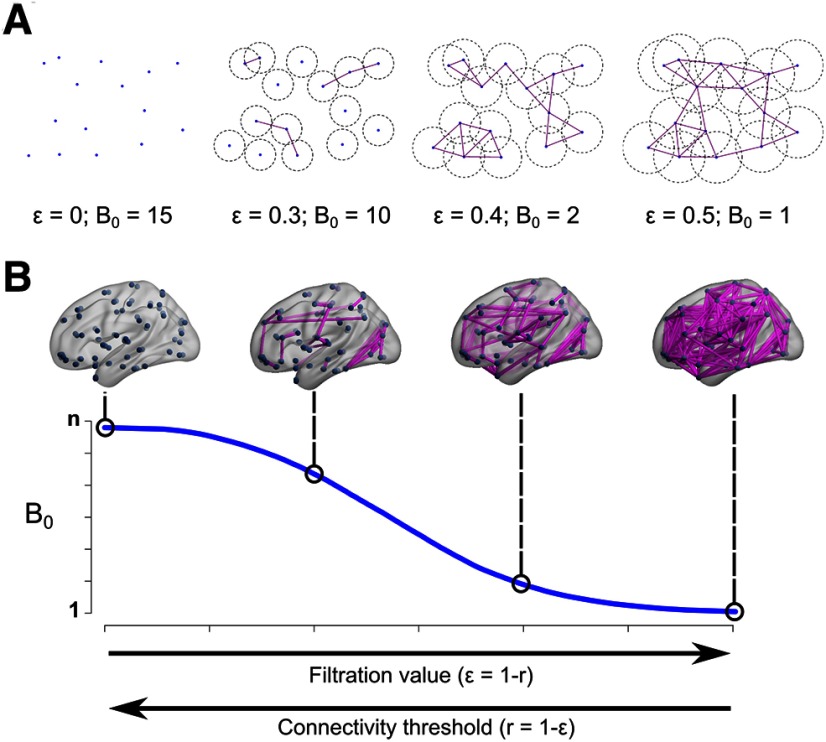
The Betti-0 curve. ***A***, Two-dimensional toy example: a set of 15 nodes, four filtration values ε, represented as the circle diameter and their corresponding Betti-0 (B_0_). ***B***, Betti-0 curve for a hypothetical brain network; each point in the curve represents the B_0_ for each filtration value. In both cases, at ε = 0 the number of components is equal to the number of nodes, *n*. As the filtration value increases, the number of components reduces, and eventually will reach a single one containing all nodes. Brain views generated with brain-net (Xia et al., 2013), *r* stands for Pearson’s correlation.

Recently, topological data analysis (TDA), has been adopted in neuroimaging as a tool to quantify and visualize the evolution of the brain network at different thresholds (Lee et al., 2011, 2017; Stolz et al., 2018; Sizemore et al., 2018, 2019; Expert et al., 2019; Santos et al., 2019). The main objective of this method is to model the network as a topological space instead of a graph (Edelsbrunner et al., 2000; Zomorodian and Carlsson, 2005), allowing the assessment of the functional connectivity matrix as a topological process instead of a static threshold-dependent representation of the network. One of the possible applications is to characterize how the isolated nodes gradually bind together into larger components (sets of connected nodes) as a function of the filtration value (connectivity threshold), until a single component is recruited. For this purpose, the number of components at a given filtration value is termed the Betti-0 (see Materials and Methods). This process is summarized in a so called Betti-0 curve (Fig. 1), which has been shown to differentiate children with developmental disorders from controls using data from positron emission tomography (PET) and defining the brain network at the group-level (Lee et al., 2011, 2012). However, it has been typically applied to brain networks defined at the group level, i.e., exploring the covariance of concatenated physiological or structural data from groups of subjects, instead of exploring the individual characteristics of the network and comparing them between groups. Furthermore, the consistency of the method across different brain segmentation schemes has not been explored.

In this work, TDA was applied to explore individual brain networks based on the resting state functional MRI (rsfMRI) of children diagnosed with ADHD and typically developing children (TDC), obtained from the publicly available ADHD-200 database (HD-200 Consortium, 2012). First, the consistency of this methodology was explored when using four different brain segmentation schemes (atlases), and then group differences were identified between ADHD and TDC groups, at the whole-brain and subnetwork levels. ADHD is a developmental disorder characterized by a lack of control of appropriate behavior and a difficulty to maintain attention (WHO, 1992; APA, 1994). Current theories propose the potential alteration of multiple functional networks and their interaction, including the default, cognitive control (fronto-parietal), dorsal and ventral attention, and salience networks (Sonuga-Barke and Castellanos, 2007; Castellanos and Aoki, 2016). Consequently, it was expected that the proposed methodology would reveal significant differences between groups among the components of these functional networks.

## Materials and Methods

### Sample

Imaging and phenotypic data from 263 participants corresponding to the New York University Child Study Center dataset were obtained from the ADHD-200 database (https://fcon_1000.projects.nitrc.org/indi/adhd200/). Subjects reported with a secondary diagnosis and/or not medication-naive status were discarded. Only those with good imaging quality and complete phenotypic information were used for subsequent analysis, resulting in a total of 182 children. Study protocols were approved by the New York University Institutional Review Boards, and after an explanation of study procedures a written informed consent from parents and assent from children were required.

Pediatric diagnosis was based on the Schedule of Affective Disorders and Schizophrenia for Children Present and Lifetime Version (KSADS-PL) and the Conners’ Parent Rating Scale-Revised, Long version (CPRS-LV). Moreover, IQ was measured with the Wechsler Abbreviated Scale of Intelligence (WASI). Inclusion in the ADHD group was based on the parent and child responses to KSADS-PL and obtaining a t-score greater or equal than 65 in any of the ADHD related indices of the CPRS-LV. TDC had ADHD summarized t-scores below 60, and lack of any DSM-IV axis-I disorders. Exclusion criteria were an IQ below 80 or any chronic medical conditions. However, phenotypic data of three ADHD datasets showed full intelligence scores below 80, while two TDC showed *t* scores >60 in the ADHD summary scale. Data from those subjects were discarded for further analysis, resulting in a final sample of 81 ADHD children (average age ± SD: 10.5 ± 2.48 years old) and 96 TDC (12.26 ± 3.07 years old; Table 1).

**Table 1:** Phenotypic information by diagnostic group

	TDC	ADHD	
Frequency	96	81	
Sex (F/M)	51/45	22/59	OR = 3.019
Age (years old)	12.26 (±3.07)	10.5 (±2.48)	*d* = 0.63
ADHD index	44.97 (±4.75)	72.78 (±8.18)	*d* = –4.26
IQ	111.27 (±13.92)	108.22 (±13.69)	*d* = 0.22
Motion (mm)	0.067 (±0.054)	0.072 (±0.041)	*d* = –0.11

Motion stands for the average RMS of the relative head motion within the scanner (computed with FSL’s MCFLIRT). Grouped effect sizes by odds ratio (OR) and Cohen’s *d* (*d*).

### Imaging acquisition

Magnetic resonance images were acquired with a Siemens Magnetom Allegra 3T scanner (Siemens Medical Solutions). Whole-brain fMRI volume images were obtained using a T2^*^-weighted echo planar imaging interleaved sequence (TR/TE = 2000/15 ms, flip angle = 90, voxel size 3 × 3 × 4 mm^3^, FOV = 240 × 192 mm^2^) with a scan duration of 6 min. Participants were instructed to remain still, close their eyes, think of nothing systematically and not fall asleep. In order to obtain an anatomic reference, high-resolution structural T1-weighted magnetization prepared rapid acquisition gradient echo (MPRAGE) images were acquired (TR/TE = 2530/3.25 ms, flip angle = 7°, voxel size 1.3 × 1.0 × 1.3 mm^3^, FOV = 256 × 256 mm^2^).

### Preprocessing

Preprocessing was implemented using FMRIB’s Software Libraries (FSL v.5.0.6; Jenkinson et al., 2012). Steps included removing the first four volumes, slice timing, head motion correction, brain extraction, regression of confounding variables, bandpass temporal filtering (0.01–0.08 Hz), and spatial normalization. Given that psychiatric and pediatric populations usually show higher in scanner motion than controls and adults (Satterthwaite et al., 2012), a rigorous confounding regression strategy was implemented to minimize head motion artifacts. Specifically, several variables were regressed out from the functional data, including the six rigid-body motion parameters, the average signal from both white matter (WM) and CSF, the derivative of these eight parameters and the square of these sixteen variables (Gracia-Tabuenca et al., 2018). In addition, to minimize the impact of physiological noise, five principal components of the signal from WM and CSF were also included as confounding variables (Behzadi et al., 2007; Chai et al., 2012). Furthermore, those volumes with a root mean square (RMS) of relative head motion >0.25 mm were also included as confounds (Satterthwaite et al., 2013). Subjects with an average RMS of relative head motion higher than 0.55 mm or <4 min of non-motion-affected data, were discarded. Eventually, each fMRI volume was registered to its corresponding T1 image with a rigid-body transformation, followed by an affine and nonlinear registration to a 2 × 2 × 2 mm^3^ children-specific template, the 4.5–18.5 years old NIHPD atlas (Fonov et al., 2011).

### Functional connectomes

For every dataset, four functional connectomes (connectivity matrices) were computed based on different brain atlases: AAL (Tzourio-Mazoyer et al., 2002), P264 (Power et al., 2011), CC200, and CC400 (Craddock et al., 2012). All of them include cerebrum and cerebellum. The first one consists of a segmentation of 116 anatomic regions, the second one is a set of 264 spherical ROIs with high reliability in both task and resting fMRI large datasets, while the last two are segmented based on functional connectivity homogeneity (with 190 and 351 nodes, respectively).

For each subject and atlas, the average fMRI signal of every defined region was extracted and then the functional connectome was computed as the Pearson’s cross-correlation between all possible pairs of regions. The reliability of the explored TDA variables along the atlases was assessed by the Kendall’s concordance coefficient (KCC).

### TDA

Typically, the brain connectome is modeled as a graph (G), which is a collection of nodes (V) and edges (E). Nodes usually represent regions of interest, while edges represent structural or functional connections between those nodes. Nevertheless, this graph can be represented as a topological space as well, in particular, the Rips complex, denoted by Rips(F,ε), where F represent the nodes (same as V) and ε, the filtration value, which is a positive number that states if two nodes in F are connected (if their distance is lower than ε). Algebraic properties extracted from this topological space are called Betti numbers, particularly, the Betti-0 number (B_0_) accounts for the number of components, i.e., the number of isolated nodes or sets of nodes connected by a sequence of edges; Betti-1 number refers to the number of cavities in the two-dimensional space between nodes, and so on (for extensive review on TDA, see Edelsbrunner et al., 2000; Sizemore et al., 2019). In this work, we focus exclusively on B_0_. If we start with a filtration value ε = 0, all nodes are disconnected, and the number of components equals the number of nodes. When ε gradually increases, some isolated nodes will connect with others and the number of components decreases. Therefore, B_0_ will diminish as the nodes gradually connect to each other as ε increases. It is possible to identify the filtration values for which there is a change in B_0_, until there is only one large component containing all the nodes. This process is summarized in the so-called B_0_ curve (Fig. 1).

Here, the distance between nodes is defined as in Lee et al. (2012), i.e., d(ξ, x_j_) = 1 − *r*(ξ, x_j_), where *r* is the Pearson’s correlation between the pair of nodes ξ and x_j_. The B_0_ curves are computed with the TDA package in R (https://cran.r-project.org/web/packages/TDA/index.html), and characterized in terms of the area under the curve (AUC), slope, and kurtosis. The AUC accounts for the overall transition from all nodes being isolated to being connected into a single component, with smaller areas suggesting that B_0_ decreases with smaller filtration values. The slope accounts for the rate of change, being all negative, lower values mean a faster transition to a single component. Finally, the kurtosis accounts for how “tailed” the distribution is with respect to the average value, with higher values meaning faster transition to a single component.

### Null model

In order to prove that the estimated parameters of the B_0_ curves are features of the brain topology, observed values were compared with those generated from a random distribution. A weighted null distribution was calculated based on the rewiring of the original connectivity matrices (Giusti et al., 2015). Specifically, 1000 permutations were run for each subject and parcellation.

### Diagnostic group inferences

Logistic regression was applied to identify significant associations between diagnostic group and the B_0_ features, including sex, age, and head motion as covariates (Eq. 1). This approach allows the calculation of the effect sizes of the orthogonal odds ratio (OR) for each term of the equation. All dimensional variables were standardized to *z* scores to be included in the model. This strategy was applied for each of the four brain atlases.

(1) group ∼ AUC + kurtosis + slope + sex + age + motion

Equation 1 expresses logistic regression model according to Wilkinson-Rogers notation (Wilkinson and Rogers, 1973); “motion” accounts for average RMS relative head motion (mm).

In addition, the same logistic regression model (Eq. 1) was implemented at intranetwork and internetwork levels, i.e., considering the seven lobes of the AAL atlas, and the thirteen functional networks defined in P264. For example, logistic regression was applied for the subset of 28 frontal lobe nodes (based on AAL atlas), referred to as the Frontal intranetwork. Then, it was applied for the frontal-parietal internetwork subset (28 + 14 nodes), and so on. Given the 28 and 91 possible combinations, for the AAL and P264, respectively, significance was set to *p* < 0.05, corrected for a non-parametric family-wise error (FWE) approach based on clusters of edges (Network Based Statistics; Zalesky et al., 2010), in which a *p* < 0.05 (bi-sided) was set for individual edges, and a null distribution of clusters was computed with 10,000 permutations.

### Code accessibility

The code/software described in the paper is freely available online at https://github.com/BrainMapINB/TDA_ADHD. Also, the code is available as Extended Data 1. Present results were computed with an Intel Core i7-4790 CPU @ 3.60 GHz × 8 with Ubuntu 18.04.3 LTS 64-bit.

10.1523/ENEURO.0543-19.2020.ed1 Extended Data 1 Code applied in the present article. Download Extended Data 1, ZIP file.

## Results

### Agreement across brain atlases

**Figure 2.**
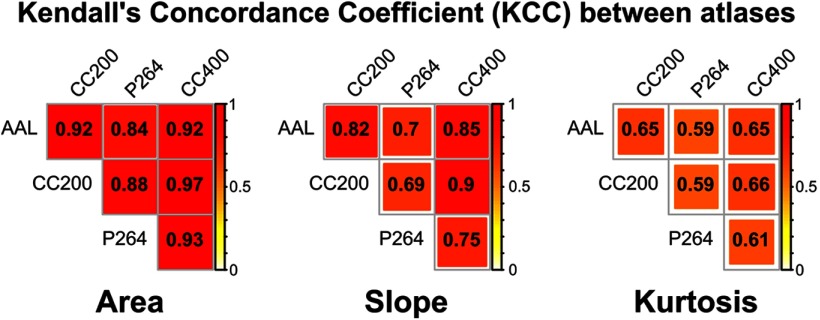
KCC between brain parcellations for the explored properties of the B_0_ curves: AUC, slope and kurtosis. KCC value is depicted in yellow-red, with *p*(KCC > 0.59) < 0.05, given two raters and 176 degrees of freedom.

The three explored properties of the B_0_ curves showed a generalized sample agreement along the four brain parcellations. Significant agreement was found considering every atlas and each feature: area (KCC = 0.87; χ^2^_176_ = 609; *p* = 4.67e-49), slope (KCC = 0.68; χ^2^_176_ = 477; *p* = 1.33e-29), and kurtosis (KCC = 0.44; χ^2^_176_ = 307; *p* = 3.65e-09). Moreover, pairwise concordance coefficient was significant for every pair of atlases and every TDA metric (Fig. 2).

### Observed versus null model

**Figure 3.**
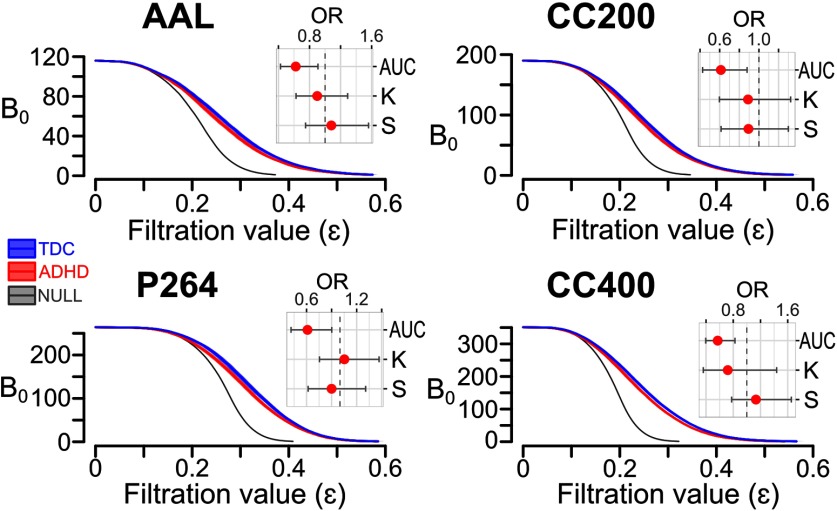
B_0_ curves for each group and brain parcellation. Group average with 95% confidence interval (CI) of the B_0_ curves. Forest plot of OR with 95% CI for the logistic regression terms of AUC, kurtosis (*K*), and slope (*S*) are depicted for each brain atlas: AAL (top left), CC200 (top right), P264 (bottom left), and CC400 (bottom right). Gray dashed line stands for OR equal to one.

When comparing observed results against those generated from the null model, B_0_ curves from the randomized data reached the single component faster than the original data (Fig. 3). Regarding the explored parameters, the AUC and slope of the null model never reach the observed values, while permuted kurtosis are close to the original values in almost half of the iterations (*p* = 0.4098).

### Whole-brain topology

**Figure 4.**
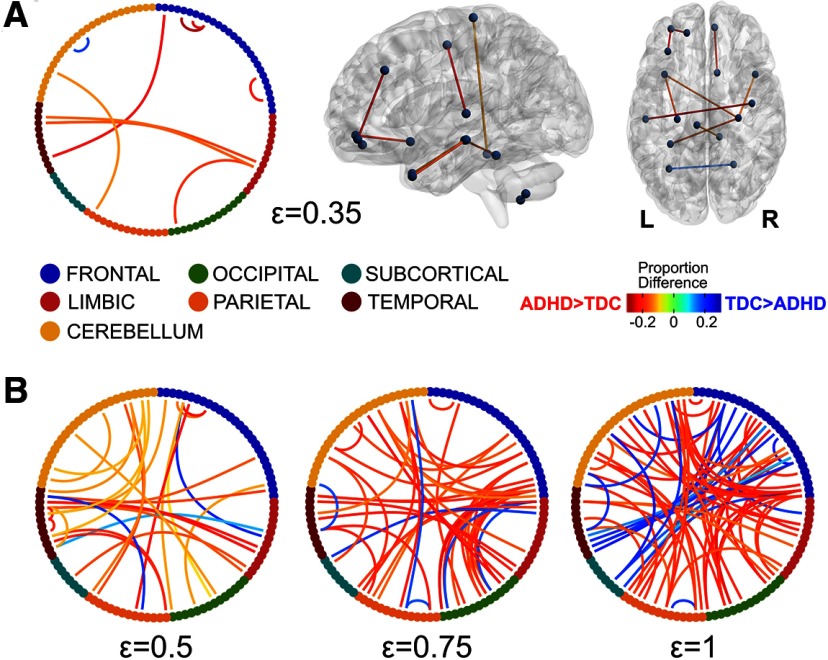
Edges with differences in the proportion of subjects between groups at ε = 0.35 (***A***) and ε = 0.5, 0.75, and 1 (***B***). Nodes from each lobe (AAL atlas) are represented with different colors in the chord diagrams. Only edges with a proportion difference at *p* < 0.01 (uncorrected) are depicted. For ε = 0.35 the edges are represented in the brain using brain-net (Xia et al., 2013). R stands for the right side of the brain.

The area under the B_0_ curves showed significantly lower odds for the ADHD group, no matter the brain atlas (AAL: OR = 0.622, *p* = 0.014; CC200: OR = 0.612, *p* = 0.008; P264: OR = 0.611, *p* = 0.013; CC400: OR = 0.572, *p* = 0.003), which means that the ADHD group has smaller AUC compared with the TDC group (Fig. 3). The area under the B_0_ curves accounts for the overall transition from all nodes being isolated to being connected into a single component, with smaller areas when B_0_ decreases faster as the filtration value increases. In other words, less AUC implies lower number of components, i.e., less segregation, which should be mediated by increased connectivity in the edges mediating the integration of components. In order to explore such edges, the proportion of subjects showing connectivity for each edge at some filtration values was compared between groups (Fig. 4). These tests showed widespread frontal short-range and cortical long-range edges being more frequently present in the ADHD group (*p* < 0.01, uncorrected; Fig. 4). No group differences were found for the slope nor the kurtosis, thus only the AUC was sensitive to differentiate both groups.

### Intranetwork and internetwork inference

**Figure 5.**
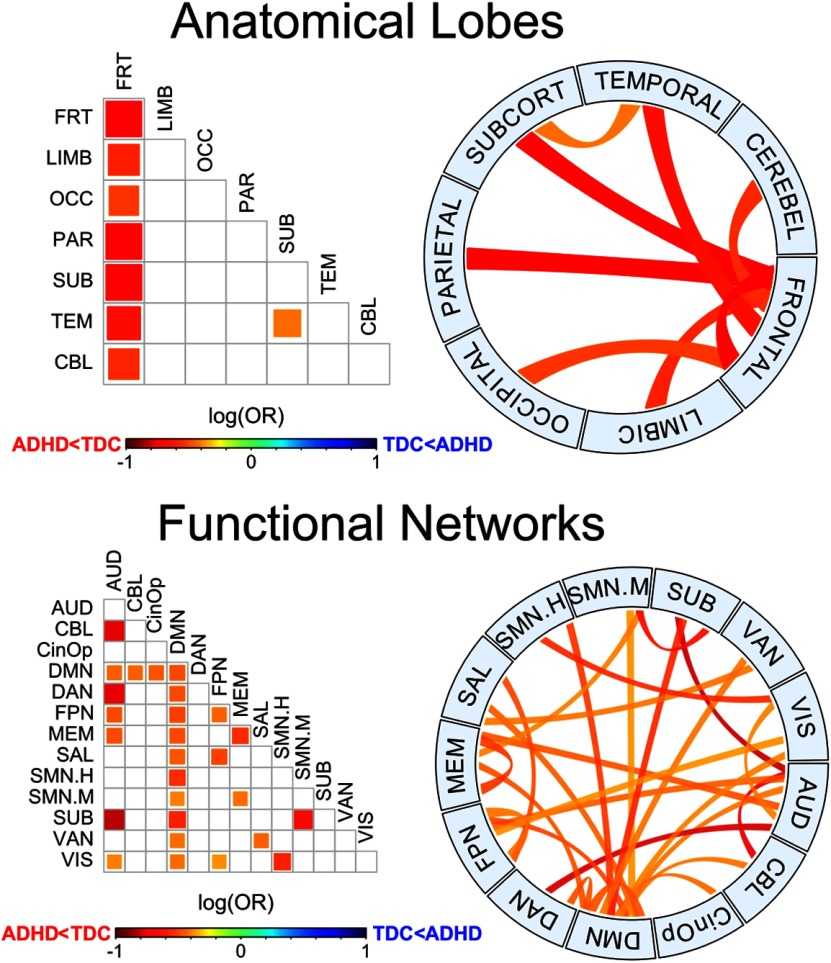
Group differences for anatomic and functional subnetworks. Pairwise plot and chord diagrams (Gu et al., 2014) of significant differences for the area under the B_0_ curves (*p*_FWE_ < 0.05) between groups. Anatomical lobes (top) are based on AAL parcellation and functional networks (bottom) are based on P264 parcellation. AUD, auditory; CBL, cerebellar; CinOp, cingulo-opercular; DMN, default mode; DAN, dorsal attention: FPN, fronto-parietal; MEM, memory retrieval; SAL, salience; SMN.H, sensory/somatomotor hand; SMN.H, sensory/somatomotor mouth; SUB, subcortical; VAN, ventral attention; VIS, visual.

Logistic regressions were performed in subsets of nodes as well, corresponding to the nodes of a single lobe or functional network (intranetwork) or the nodes of two networks (internetwork), according to the lobular and the functional parcellation of the AAL and P264 atlases, respectively. Resulting patterns of the AUC were significant after FWE correction, *p*_FWE_ = 0.0026 and *p*_FWE_ = 0.0147, respectively. Anatomical subsets included the intranetwork and every possible internetwork subset including the frontal lobe plus the temporal-subcortical interaction, and in every case the ADHD group showed a lower area compared with the TDC (Fig. 5). These results demonstrate a widespread decreased segregation of the brain network in the ADHD group, particularly involving the frontal lobe. When considering the functional systems in the P264 atlas, notably, all the subsets of nodes that included the default mode network (DMN) also showed smaller areas for the ADHD group, but other intranetwork and internetwork subsets showed similar patterns (Fig. 5).

## Discussion

In this work, methods from TDA were applied to explore the topology of the brain network as a function of the filtration value (i.e., the connectivity threshold). Resulting B_0_ curves were characterized in terms of three parameters: AUC, slope, and kurtosis; and compared between ADHD and TDC. The application of this model to a pediatric sample showed that the AUC was significantly lower for the ADHD group, both at the whole-brain and at the subnetwork level. These results showed decreased functional segregation in the ADHD group, mainly involving the frontal lobe and the DMN.

The B_0_ curves were characterized in terms of three parameters, the AUC, slope, and kurtosis. Pairwise agreement between brain parcellations was high for the AUC (KCC range: 0.84–0.97) and the slope (KCC range: 0.69–0.9), and medium to low for the kurtosis (KCC range: 0.59–0.66). These results suggest that this methodology is consistent among different parcellation schemes, especially for the area under the B_0_ curve. In addition, considering that the B_0_ features do not depend on a particular connectivity threshold, but instead explore all the filtration values with a change in the topology of the network, this methodology contributes to provide a complete picture of the brain network, overcoming one of the main limitations of other approaches. Taken together, these are potentially important advantages that may complement other methods applied to brain networks, such as graph theory, which has been shown to be highly dependent on the brain parcellation scheme (Wang et al., 2009a; Chen et al., 2018; Doucet et al., 2019), and on the selection of a connectivity threshold or connectivity cost (van den Heuvel et al., 2008; Fornito et al., 2010; Tomasi and Volkow, 2010; Termenon et al., 2016; Gracia-Tabuenca et al., 2018).

The area under the B_0_ curve was significantly lower for the ADHD group, both at the whole-brain network and at the subnetwork level, being strikingly significant for the interactions involving the frontal lobe and the DMN. As mentioned above, the AUC accounts for the overall transition from all nodes being isolated to being connected into a single component, with smaller areas suggesting that B_0_, the number of components, decreases faster as the filtration value increases. Such differences in B_0_ for a given filtration value are mediated by edges that bind together previously split components, which results from increased connectivity in some edges mediating the integration into larger components. Taken together, the results here presented can be interpreted as higher functional connectivity within the connectome and specific subnetworks in the ADHD group, especially those involving the frontal lobe and the DMN. Previous evidence has also suggested increased functional connectivity in a variety of regions of the frontal lobe in ADHD (Tomasi and Volkow, 2012; Hoekzema et al., 2014; Mostert et al., 2016), as well as fronto-occipital (Cocchi et al., 2012) and fronto-subcortical connections (Cocchi et al., 2012; Tomasi and Volkow, 2012), particularly those associated with reward and motivation (Tomasi and Volkow, 2012). Our results showed a similarly widespread pattern in several functional subnetworks, mainly the DMN, but also attention, salience, fronto-parietal, and auditory nodes, among others (Fig. 5). These results provide the basis to infer the potential functional systems being affected in ADHD, being consistent with the current theories involving such networks (Castellanos and Aoki, 2016), particularly with the DMN interference hypothesis, which is based on the findings of altered interactions between the DMN and networks involved in top-down executive control (Fox et al., 2005; Fox and Raichle, 2007; Kelly et al., 2008; Elton et al., 2014; Hoekzema et al., 2014; Castellanos and Aoki, 2016; Bos et al., 2017; Qian et al., 2019).

Previous studies have reported a myriad of differences in network properties between ADHD and TDC participants. At the whole-brain level, higher functional segregation and lower functional integration in ADHD subjects compared with controls have been reported (Wang et al., 2009b; Lin et al., 2014), although other groups did not reproduce those results (Cocchi et al., 2012; Sato et al., 2013). Since the decreased area under the B_0_ curves could be interpreted as higher integration and lower segregation of isolated components, our results seem to be contradictory to the aforementioned ones. Nevertheless, the previous studies explored connectivity costs higher than 10%, which according to Lin et al. (2014) would correspond to filtration values higher than ε = 0.5, when most of the subjects actually exhibit a single component (Fig. 3). Therefore, these results are actually complementary, given that B_0_ curves consider a wider range of connectivity thresholds, rarely explored with graph theory. Indeed, when exploring the edge-wise proportions between groups at different connectivity thresholds (Fig. 4), the ADHD group showed consistently widespread increases compared with the TDC. However, at lower connectivity thresholds (higher filtration values), the ADHD group showed decreased proportion of edges in several interactions, mainly including the frontal, temporal, subcortical, and cerebellar regions, which seem consistent with previous reports of decreased connectivity in ADHD (Tomasi and Volkow, 2012; Di Martino et al., 2013; Elton et al., 2014). These results evidence that the static representation of the network changes as a function of the connectivity threshold, therefore an approach that takes into account wider threshold ranges should provide better insights into the neurophysiological substrate of ADHD.

As far as we are concerned, only two previous studies have explored B_0_ in ADHD brain networks (Lee et al., 2011, 2012), using fludeoxyglucose PET (FDG-PET) and interregion covariation at the sample level, qualitatively reporting higher number of components for the ADHD group compared with the TDC. Such findings seem to be opposite to the results here presented; however, methodological differences prevent direct comparisons between results. First, time-scales are significantly different, with the FDG-PET scans reflecting the glucose uptake occurring during several minutes; in contrast, rsfMRI reflects variations in blood oxygenation during tens of seconds. Furthermore, FDG-PET connectivity matrices reflect interregion covariation of (long-term) glucose metabolism across subjects, while rsfMRI connectivity matrices reflect interregion covariation (within seconds) within the same subject and later compared between groups. Overall, both methodologies potentially reflect complementary aspects of the functional connectomes in ADHD.

## Conclusion

In summary, the present study showed a robust and informative implementation of TDA in functional connectomics. The results exhibited significant differences for the brain topology of children with ADHD, both at the whole-brain network and at the functional subnetwork level, particularly involving the frontal lobe and the DMN. Therefore, this approach may contribute to identifying the physio-pathology of neurodevelopmental disorders, complementing other connectomics methods by exploring a larger connectivity range and reducing the bias of selecting a fixed threshold.

## Synthesis

Reviewing Editor: Julie Bakker, University of Liege Decisions are customarily a result of the Reviewing Editor and the peer reviewers coming together and discussing their recommendations until a consensus is reached. When revisions are invited, a fact-based synthesis statement explaining their decision and outlining what is needed to prepare a revision will be listed below.

Synthesis: the paper has been re-reviewed by one reviewer and myself. We both feel that the authors have addressed all the previous concerns raised in a satisfactorily manner.
